# Within the Brain: The Renin Angiotensin System

**DOI:** 10.3390/ijms19030876

**Published:** 2018-03-15

**Authors:** LaDonya Jackson, Wael Eldahshan, Susan C. Fagan, Adviye Ergul

**Affiliations:** 1 Program in Clinical and Experimental Therapeutics, College of Pharmacy, University of Georgia, Augusta, GA 30912, USA; lajackson1@augusta.edu (L.J.); weldahshan@augusta.edu (W.E.); sfagan@augusta.edu (S.C.F.); 2 Department of Neurology, Medical College of Georgia, Augusta University, Augusta, GA 30912, USA; 3 Department of Physiology, Medical College of Georgia, Augusta University, Augusta, GA 30912, USA; 4 Charlie Norwood Veterans Affairs Medical Center, Augusta, GA 30904, USA

**Keywords:** angiotensin, angiotensin AT1 receptor, angiotensin AT2 receptor, mas receptor, mas-related-G protein coupled MrgD receptor, inflammation, microglia, vascular cognitive impairment (VCI), alzhemiers disease (AD), parkinson’s disease (PD), aging

## Abstract

For many years, modulators of the renin angiotensin system (RAS) have been trusted by clinicians for the control of essential hypertension. It was recently demonstrated that these modulators have other pleiotropic properties independent of their hypotensive effects, such as enhancement of cognition. Within the brain, different components of the RAS have been extensively studied in the context of neuroprotection and cognition. Interestingly, a crosstalk between the RAS and other systems such as cholinergic, dopaminergic and adrenergic systems have been demonstrated. In this review, the preclinical and clinical evidence for the impact of RAS modulators on cognitive impairment of multiple etiologies will be discussed. In addition, the expression and function of different receptor subtypes within the RAS such as: Angiotensin II type I receptor (AT1R), Angiotensin II type II receptor (AT2R), Angiotensin IV receptor (AT4R), Mas receptor (MasR), and Mas-related-G protein-coupled receptor (MrgD), on different cell types within the brain will be presented. We aim to direct the attention of the scientific community to the plethora of evidence on the importance of the RAS on cognition and to the different disease conditions in which these agents can be beneficial.

## 1. Introduction

Renin angiotensin system (RAS) research has a long and rich history dating back to the discovery of renin in 1898 [1]. In the past 120 years, other components of this potent system including angiotensinogen, angiotensin converting enzyme (ACE) isoforms, angiotensin peptides, and their cognate receptors have been discovered and pharmacological tools that modulate this system at many levels have been developed [2]. This complex system has important physiological functions in regulation of water and electrolyte balance, systemic vascular resistance, blood pressure and cardiovascular homeostasis. However, its chronic activation can cause oxidative stress, endothelial dysfunction and inflammation resulting in many pathological conditions ranging from hypertension, kidney disease and heart failure [3]. As such, ACE inhibitors and angiotensin II type 1 (AT1R) receptor blockers (ARBs) have been evaluated in many clinical trials. Recent evidence suggests that inhibition of this system may be beneficial in attenuating cognitive deficits observed in aging, Alzheimer’s Disease (AD), Parkinson’s Disease (PD), Vascular Cognitive Impairment (VCI) and Post-Stroke Cognitive Impairment (PSCI) [4,5,6].

While originally it was considered to be a systemic (endocrine) system, subsequent research discovered that in addition to “classical” systemic RAS, there is a “local” RAS in many tissues, including the brain. In this system, angiotensin II (Ang II) remains to be the most studied vasoactive substance. In addition to vascular and renal actions, Ang II has direct effects at the cellular level impacting cell survival, differentiation and even inflammation [5,7,8]. In this special issue on “Roles of Cardiovascular Active Substances and Cellular Events in the Homeostasis of Cardiovascular Systems”, we will focus on the RAS within the brain and review its impact on cognitive function.

## 2. Central Renin Angiotensin System

Within the brain, there are peripheral and central angiotensinergic pathways. The main peripheral pathway is the forebrain pathway which integrates the circumventricular organs (CVOs) that surround the third and fourth ventricles and consists of fenestrated capillaries to allow for the peripheral access of RAS components [9]. Although structures along the forebrain pathway have access to peripheral RAS components, the majority of brain regions do not. Unlike local RAS production in other organs, the blood–brain barrier (BBB) restricts peripheral RAS components from accessing the majority of brain regions, making the local synthesis of cerebral RAS essential. The main central angiotensinergic pathway connects the hypothalamus and medulla and is the primary contributor of locally synthesized angiotensin [9,10,11]. To supplement this, RAS components are also synthesized in various other brain regions. Both peripheral and central RAS production contributes significantly to cardiovascular homeostasis. AT1R is classically known for its presence on endothelial cells and its role in vasoconstriction, and AT2R for its role in vasodilation and can be further reviewed here [9]. For the purpose of this review, we will focus on the impact of the central RAS on cognitive function. We will focus on its cell specific actions within the cortex, hippocampus and basal ganglia, as they are key structures in the development of AD, PSCI, VCI and PD.

### 2.1. Angiotensin Ligands and Peptidases

**Figure 1:**
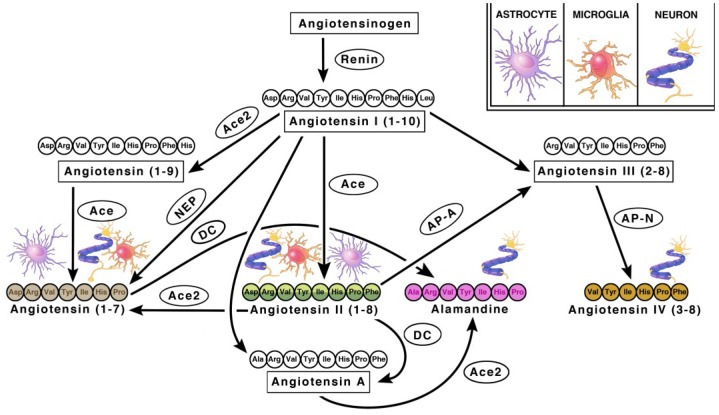
Schematic Diagram Depicting The RAS Components Within The Brain. Various brain cells possess different members of the RAS system which allows for the local synthesis and biological actions of angiotensin in the brain. The legend indicates the cells depicted within each illustration (astrocytes, neurons, and microglia). Enzymes convert precursor angiotensin fragments into neuroactive forms. Enzymes are indicated by circles. Neuroactive forms include: Angiotensins II, III, IV, (1–7), and Alamandine. The main biologically active forms are indicated with colored amino acid sequences. Abbreviations: angiotensin-converting enzyme (ACE) and (ACE2), aminopeptidase A (AP–A), aminopeptidase N (AP–N), neutral endopeptidase (NEP), decarboxylase (DC).

**Figure 2:**
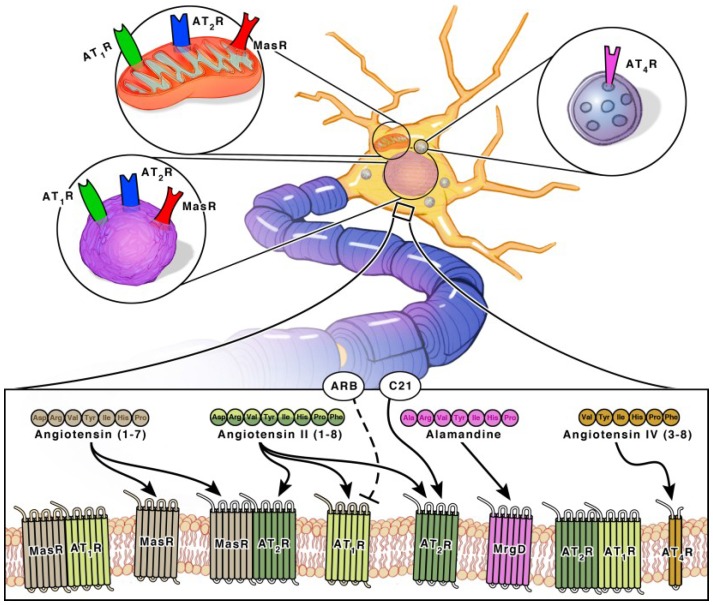
Schematic Diagram Depicting Neuronal Plasma Membrane and Intracellular RAS Signaling. Angiotensins II, IV, (1–7), and Alamandine are the main neuroactive forms and their signaling is indicated by arrows leading to their receptors. Receptors can form heterodimers. In the case of the MasR/AT2R heterodimer, arrows leading to the receptor from its ligands indicate its functionality. The AT2R/AT1R and MasR/AT1R heterodimers are inactive, indicated by an absence of arrows. Angiotensin (1–7) binds MasRs with the highest affinity, but can also bind AT2Rs and MrgDs. Angiotensin II binds AT1Rs and AT2Rs with the highest affinity. Alamandine binds MrgDs with the highest affinity. Angiotensin IV binds AT4Rs with the highest affinity, but can also bind AT1Rs. Angiotensin III is not depicted here since it is not a main neuroactive peptide, it binds AT2Rs with the highest affinity, but can also bind AT1Rs. Receptors can be located on the plasma membrane or intracellularly. Intracellular locations include the nucleus, mitochondria and neurosecretory vesicles. Therapeutics such as Angiotensin II type I receptor blockers (ARBs) and the AT2R agonist, Compound **21** (C**21**) can be used to modulate receptor signaling.

Angiotensinogen is the precursor peptide to angiotensin. Centrally, over 90% of angiotensinogen is produced within astrocytes and constitutively secreted for conversion into various neuroactive peptides [11,12]. It is also produced by neurons, where it can be secreted or remain intracellularly [11,12]. The neuroactive peptides are able to bind to their cognate receptors on various cell types to induce receptor signaling [11,12]. There are four main neuroactive angiotensin peptides: Ang II, Ang IV, Ang (1–7) and Alamandine (Figure 1 and Figure 2). To produce Ang II, angiotensinogen is cleaved into Ang I by renin, then further processed by ACE, an infamous peptidase. Ang II is the main angiotensin effector peptide and it binds two major receptors, AT1R and AT2R (Figure 2). Ang II can also be further converted to Ang III, then Ang IV by aminopeptidases (i.e., AP-N). Ang III binds AT1Rs at a lesser affinity than Ang II, and AT2Rs at a higher affinity than Ang II [13]. Ang IV binds AT4Rs and, when in high concentrations, it can also bind AT1Rs. To produce Ang (1–7), Ang I or II can be cleaved by ACE2, an isoform of ACE recently discovered in 2000, and then bind Mas receptors (MasRs) [14,15]. It is also capable of cleaving Ang I from Ang (1–9) at a lesser efficiency. Ang (1–9) can then be converted to Ang (1–7) by ACE to bind MasRs [16]. Ang (1–7) can additionally bind AT2Rs with small affinity. Ang (1–7) can also bind Mas-related-G protein coupled receptors (MrgDs) [17]. MrgDs are a recently discovered addition to the angiotensin system. The main ligand for this receptor is Alamandine, an Ang (1–7) analog that can be formed by the decarboxylation of Ang (1–7). Alamandine can also be formed by the decarboxylation of Ang II into an Ang A precursor, an Ang II analog. ACE2 can then cleave Ang A into Alamandine, which can bind MrgDs. A schematic of the enzymes and ligands can be found in Figure 1, while the main neuroactive peptides and their cognate receptors can be found in Figure 2. As detailed above, the angiotensin system is a vast and complex network of ligands and their affinities to various receptors. For the purpose of this review, we will primarily focus on the effects of receptor specific signaling and location.

### 2.2. Angiotensin Receptors and Peptidases

**Figure 3:**
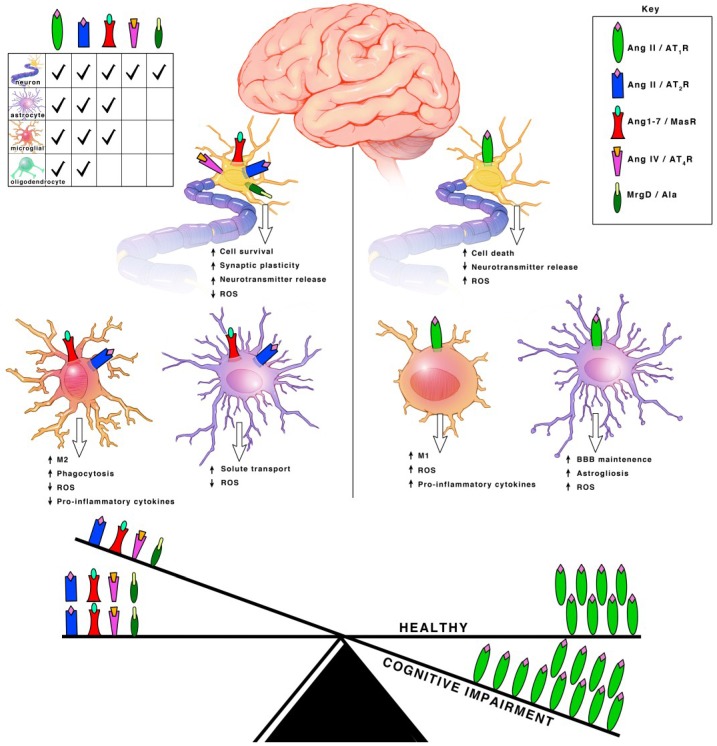
Schematic Representation of The Biological Actions of The RAS System In The Brain. Ras Components And Their Cognate Receptors Are Depicted In The Figure Key. Under physiological conditions, RAS components such as Ang (1–7), Ang II and Ang IV and their cognate receptors MasR, AT2R and AT4R that mediate beneficial effects to improve cognition are in abundance in different cell types depicted in the table. In conditions such as aging, and disease states such as Alzheimer’s Disease (AD), Parkinson’s Disease (PD), Vascular Cognitive Impairment (VCI) and Post-Stroke Cognitive Impairment (PSCI), Ang II/AT1R axis predominates and exacerbates the development of cognitive impairment, depicted by the seesaw.

Ang II can bind both the AT1R and AT2R. AT1Rs are G-protein coupled receptors (GPCRs) located on neurons, astrocytes, oligodendrocytes and microglia of the cortex, hippocampus, and basal ganglia [18] (Figure 3). ACE is responsible for converting Ang I into Ang II. Even though Ang II can bind two different receptors, ACE upregulation specifically leads to increased AT1R activation. Hyperactivation of AT1R signaling and upregulation of ACE expression is well known for its role in vasoconstriction but within the brain, specifically exacerbates cognitive impairment [19], cell death [20,21] and inflammation [18].

Both AT2Rs and MasRs are also GPCRs located on neurons, astrocytes and microglia of the cortex, hippocampus, and basal ganglia [22]. MrgDs are less studied but they are also GPCRs and are located on neurons [23,24]. AT2Rs, MasRs, MrgDs and ACE2 are known for their roles in vasodilation, but within the brain they specifically enhance cognition, cell survival and have both antioxidant and anti-inflammatory properties [18,19,25,26,27] (Figure 3). The AT2R, MasR and MrgD pathways in interdependent. ACE2 facilities the formation of ligands for MasRs and MrgDs. AT2R activation increases ACE2 expression [28]. When AT2Rs are knocked out, there is a decline in MasRs and ACE2 mRNA, protein and activity [28]. Additionally, AT2Rs and MasRs can form heterodimers that functionally depend on each other [29]. AT2Rs and MasRs can also each heterodimerize with AT1Rs, forming AT2R/AT1R and MasR/AT1R heterodimers to directly antagonize and inactivate AT1Rs, leading to a decrease in AT1R signaling pathways [27] (Figure 2). ACE2 significantly increases the formation of these AT2R/AT1R and MasR/AT1R heterodimers [16]. Apart from the formation of inactive heterodimers, ACE2 upregulation also results in the overall downregulation of AT1Rs. This suggests a possible effect on AT1R transcription and/or internalization [16,30,31]. Upregulation of AT1R activation also reciprocally leads to downregulation of ACE2 [16,30,32]. This combined suggests a synergistic interplay between the receptors and enzymes to maintain a delicate balance for cognition within a healthy brain.

The AT4R is unique in that it is not a GPCR, but an insulin-regulated aminopeptidase (IRAP). A type II aminopeptidase membrane protein that is located on neurons in the cortex, hippocampus, and basal ganglia [28,33] (Figure 2). Unlike the other receptors, AT4Rs may be restricted to neurons. AT4R activation improves cognition, cell signal transmission, synaptic remodeling and has both antioxidant and anti-inflammatory properties [34,35].

### 2.3. Renin in the Brain

Renin is responsible for initiating the cascade of angiotensin peptide formation by cleaving angiotensinogen into angiotensin I. Classical renin is the active form derived from the cleavage of pre-pro segments and is abundant in the peripheral circulation, but it has a low concentration in the brain [11,12]. Renin has been identified within neurons and astrocytes [11]. Within neurons, an intracellular and secreted renin has been identified [12]. Secreted renin is in the form of prorenin. The prorenin has a high concentration in the brain and binds to prorenin receptors (PRRs) at a higher affinity than renin. PRRs also have a high concentration within the brain, and upon activation by prorenin or renin, PRR signaling leads to angiotensinogen cleavage. Over activation of renin/prorenin signaling leads to cognitive impairment through the activation of the Ang II/AT1R axis [11,12,13]. We will focus on the angiotensin peptides and receptors. The cell-type-specific signaling and cellular locations of the angiotensin receptors will be further elucidated throughout this review. We aim to connect preclinical and clinical observations in an effort to identify RAS targets for therapeutic interventions in cognitive disorders.

## 3. Angiotensin Receptor Functions within Each Cell Type

### 3.1. Neurons

Each angiotensin receptor is expressed on the cell surface of neurons. Neurons additionally have an intracellular angiotensin system (Figure 2). AT1Rs, AT2Rs and MasRs are located at the mitochondrial and nuclear levels [12,28,36,37,38] (Figure 2). Since the discovery of MrgDs is relatively new, information on its possible intracellular signaling system has not been investigated. Although the majority of the angiotensin precursor, angiotensinogen, is derived from astrocytes, neurons can additionally synthesize angiotensinogen intracellularly. They also contain PRR, ACE and ACE2 transmembrane receptors. Intracellular neuroactive angiotensin peptides can bind intracellular AT1Rs, AT2Rs and MasRs (Figure 2). The presence of receptors at the mitochondrial level involves regulation of oxidative stress [28,37]. The presence of receptors at the nuclear level also involves regulation of oxidative stress, as well as the transcription and trafficking of additional receptors types [28,39]. AT4Rs are also located intracellularly at the cytosolic level. They are mostly present in neurosecretory vessels, consistent with their role as IRAP receptors which participate in GLUT4-mediated glucose uptake and are translocated to the cell surface upon activation [40].

#### 3.1.1. Angiotensin II Type I Receptor (AT1R) and Angiotensin Converting Enzyme (ACE)

Hyperactivation of AT1R and ACE signaling in neurons exacerbates cognitive impairment, cell death, and inflammation [18]. Extracellular AT1R signaling induces the NADPH oxidase 2 (NOX2) axis to lead to increased reactive oxygen species (ROS) production resulting in increased oxidative stress [28]. AT1R activation can also lead to the nuclear translocation of the Ang II/AT1R complex and an increase in angiotensinogen, renin, and prorenin/renin receptor mRNA, leading to an increase in the synthesis of intracellular Ang II to bind AT1Rs [29]. As a compensatory mechanism, nuclear AT1R activation increases AT2R expression leading to an increase in AT2R trafficking to the mitochondria and the cell surface [28]. Nuclear AT1R additionally leads to the upregulation of Ang (1–7). Ang (1–7) primarily binds MasRs, but additionally binds AT2Rs and MrgDs [28]. The upregulated Ang (1–7) and the increased AT2R expression are compensatory mechanisms that are blunted in aging and in cognitive disorders [18]. Nuclear AT1R signaling activates the NOX4 axis, also leading to increased ROS production. Mitochondrial AT1R activation is the major site for angiotensin-induced ROS production within neurons, also via the NOX4 axis [28].

This cascade of oxidative stress activated by AT1R and ACE signaling is a major mechanism which can exacerbate cell death in regions key to cognitive function such as the cortex, hippocampus and basal ganglia. In rodents, AT1R activation led to the expansion of cortical and hippocampal cholinergic and non-cholinergic cell death after ischemic injury [21]. Upregulated ACE expression reduced acetylcholine release from cholinergic neurons [41,42]. Cholinergic cell death and dysfunction is a common feature observed across cognitive disorders [43]. Within the basal ganglia of rodents and non-human primates, AT1R upregulation induced dopaminergic cell death and dysfunction [18,44,45]. The AT1R mediated upregulation of oxidative stress led to the release of pro-inflammatory cytokines and inflammation to further exacerbate cell death [18,44,45]. The inflammatory cascade is further compounded by the activation of pro-inflammatory microglia (reviewed under microglia), ultimately leading to impaired cognition.

#### 3.1.2. Angiotensin II Type II Receptor (AT2R)

AT2R activation facilitates cognition, cell survival and has both antioxidant properties and anti-inflammatory properties [18,19,25,26,27]. Cell surface AT2R can lead to the activation of pathways such as Src Homology Region 2 Domain-Containing Phosphatase-1 (SHP–1), serine-threonine phosphatase (PP2A) and Peroxisome Proliferator-activated Receptors (PPARγ) to promote cell survival and counteract the AT1R activation [46,47,48]. Nuclear AT2R activation leads to increased nitric oxide (NO) production [49]. AT2R induced NO signaling leads to decreased firing rates and hyperpolarization via decreased activity of T-type calcium channels and delayed rectifier potassium channels as a neuroprotective mechanism after brain injury [46]. In mitochondria, AT2Rs are much more abundant than AT1Rs and are downregulated with age, while AT1Rs are upregulated. The expression of mitochondrial AT2R is upregulated further upon oxidative stress and decreases mitochondrial respiration to relieve oxidative stress through NO production [28].

The neuroprotective mechanism activated by AT2R alleviated impaired cognition in regions key to cognitive function such as the cortex, hippocampus and basal ganglia. In rodents, AT2R activation increased VEGF production and enhanced survival of cortical neurons to improve neurological deficits after ischemic injury [25]. Within the hippocampus, reduced AT2R activation actually induced dendritic spine abnormalities and spatial memory deficits [26]. Within the basal ganglia, AT2R expression was reduced in conjunction with dysfunctional signaling of dopaminergic neurons in animal models of PD. In general, dysfunctional AT2R signaling leads to increased AT1R mediated NOX activation and ROS production [36,50], all of which can lead to impaired cognition.

#### 3.1.3. Mas Receptor (MasR) and ACE

Similar to AT2R, MasR and ACE2 signaling facilitates cognition and cell survival, and has both antioxidant and anti-inflammatory properties [16,51]. Cell surface MasRs are highest in abundance. Cell surface, nuclear, and mitochondrial MasRs increase NO to decrease mitochondrial respiration, modulate superoxide levels and block the AT1R NOX mediated increase in superoxide [28]. Nuclear MasRs additionally decrease the expression of AT2R mRNA without effecting the expression of AT1R mRNA [28]. As stated previously, nuclear AT2R activation leads to increased NO and decreased neuronal firing rates as a neuroprotective mechanism after brain injury. It may be possible that this synergy exists to limit the amount of NO produced and to restore neuronal firing rates. Interestingly, ACE2 is present with highest abundance at the mitochondrial membrane and as stated previously, increased ACE2 leads to increased inactivation of AT1R through the formation of MasR/AT1R and AT2R/AT1R heterodimers. This may exist as a mechanism to limit AT1R induced ROS production at the level of the mitochondria. AT2Rs are more abundant than MasRs at the mitochondrial membrane, but due to the increased ACE2, its substrate, Ang (1–7), is more abundant than Ang II [28]. Ang (1–7) primarily binds MasRs, but also binds AT2Rs and MrgDs. The high presence of Ang (1–7) may allow for increased activation of all three receptors. Since mitochondrial AT1Rs are the main contributors to ROS production, the increased activation of all three receptors, combined with the ACE2 mediated inactivation of AT1Rs, may be the key regulatory pathway to limit oxidative stress in a heathy brain. Interestingly, AT2Rs and MasRs decline with age, while AT1Rs increase [18]. This suggests that the aging process may modulate RAS.

The aging process and the progression of cognitive disorders may either induce or be exacerbated by ROS dysfunction through disturbance of angiotensin receptor balance, to impact cognition. In rodents, MasRs were upregulated in the area surrounding the infarct cortex 6 h following ischemia and peaked after 24 h [52]. MasR activation and ACE2 overexpression both reduced iNOS and the production of pro-inflammatory cytokines: IL-1β, IL-6, TNFα in the peri-infarct cortex [20,47,49,52]. In the hippocampus and basal ganglia, MasR activation promoted cell survival and healthy synapse formation in rodent, non-human primate and humans [28,49]. As a result, improved cognition, as measured through Morris Water Maze performance, was observed [28,49]. The increased NO and the decreased ROS and pro-inflammatory cytokine release is a potential mechanism by which the cell survival, healthy synapse formation and enhanced cognition were achieved. Dysfunctional MasR signaling leads to increased AT1R mediated NOX activation and ROS production, all of which can lead to impaired cognition [36,50].

#### 3.1.4. Angiotensin IV Receptor (AT4R)

AT4R signaling improves cognition, cell signal transmission and has anti-inflammatory properties [35]. AT4Rs are IRAPs that mainly reside on neurosecretory vesicles with GLUT4. Upon AT4R activation, the receptor and GLUT4 are translocated to the cell surface. AT4R signaling operates by one of four mechanisms. First, Ang IV binding to the AT4R enhances neuronal glucose uptake and competitively inhibits IRAP to prevent metabolism of other neuroactive peptides such as Ang III, oxytocin, vasopressin, met-enkephalin which have been implicated in learning and memory [53]. Interestingly, Ang III is the precursor molecule to Ang IV, suggesting an intrinsic negative feedback mechanism for AT4R signaling. When this balance is disturbed and Ang IV is overexpressed, it binds and activated AT1Rs. Demonstrating the need for a well-balanced system. The fact that the blockage of IRAP alone can supplement but cannot produce the same effects on learning and memory as AT4R signaling, points to the fact that there are additional mechanisms at play, apart from IRAP inhibition [54]. Second, Ang IV increases intracellular calcium to enhance NO synthase (NOS) to modulate superoxide production in a manner similar to AT2Rs and MasRs [51]. Third, Ang IV increases Ca^2+^ levels via L-type voltage-dependent calcium channel activation, leading to post-synaptic modulations to enhance synaptic transmission and long-term potentiation (LTP) [55]. Lastly, similar to the effects of glucose, Ang IV enhances acetylcholine synthesis and release [51].

AT4Rs and cholinergic neurons are closely associated in the cortex and hippocampus [56], suggesting a main role in learning and memory. In rodents, AT4R signaling in the hippocampus activated acetylcholine and glutamate release. AT4R signaling also increased spinal width, spinogenesis, and induces LTP [57,58]. Within the basal ganglia, AT4R signaling induced release of dopamine [53,56]. The dysfunctional AT4R signaling could potentially lead to increased AT1R mediated NOX activation and ROS production via 2 mechanisms. Either via enhanced AT1R stimulation when Ang IV is overexpressed, or via reduced NOS when Ang IV/AT4R signaling is reduced. Balanced Ang IV can lead to increased Ang III, which binds to AT2Rs with the highest affinity to further promote NOS mediated benefits. Dysregulated AT4R signaling can lead to decreased Ang III/AT2R signaling, and other neuroactive substances beneficial to cognition. Lastly, AT4R dysfunctional AT4R signaling may lead to impaired neurotransmitter release and synaptic dysfunction, all of which can lead to impaired cognition.

### 3.2. Microglia

Microglia are the resident immune cells of the brain and the principle mediators of inflammatory responses. In a healthy brain, neurons secrete immunosuppressive proteins that keep microglia in an inactive state where their primary role consists of scanning for disturbances. Upon activation, they exist as two broadly classified phenotypes referred to as ‘M1’ or ‘M2’. Although M2 consists of many sub-types, overall, they promote immune suppression, injury resolution and participate in phagocytosis and matrix maintenance. M1 on the other hand, promotes the release of pro-inflammatory cytokines and recruitment of peripheral inflammatory cells. AT1Rs, AT2Rs and MasRs are all expressed on the cell surface of microglia but additionally have an intracellular angiotensin system with AT1Rs, AT2Rs and MasRs located at the mitochondrial and nuclear levels [59,60]. The presence of receptors at the mitochondrial level involves regulation of oxidative stress [28,37]. The presence of receptors at the nuclear level also involves regulation of oxidative stress, through the increased transcription of pro-inflammatory cytokines, and can induce mitochondrial DNA damage [28,61]. Like neurons, nuclear signaling in microglia can induce the transcription and trafficking of additional receptor types that warrants further future investigation.

#### 3.2.1. AT1R and ACE

While AT1Rs on microglia are undetectable under non-pathologic conditions, they are upregulated as part of a pro-inflammatory response in parallel with M1 microglia [18]. Activation of cell surface or intracellular AT1R signaling exacerbates cognitive impairment, cell death, and inflammation through the activation of the NOX axis and ROS production, as described in neurons [28,38,61]. Additionally, nuclear activation of AT1R leads to the upregulation of AT1Rs and a shift toward an M1 phenotype.

M1 microglia constitutively produce pro-inflammatory cytokines that can exacerbate ROS production and cell death in regions key to cognitive function such as the cortex, hippocampus and basal ganglia. AT1R upregulation in cortical and hippocampal M1 microglia is linked to neuroinflammation and cognitive impairment in rodents through a TLR4-dependent mechanism [62]. While within the basal ganglia, AT1R signaling exacerbated dopaminergic cell death similar to the upregulation observed in neurons, specifically through the activation of NOX signaling [53,54,55]. Taken together with the fact that both AT1R and ACE inhibition led to decreased ROS, pro-inflammatory production and increased M2 activities such as phagocytosis, this suggests that the AT1R mediated activation of M1 pro-inflammatory may be a mechanism that exacerbates cell death and inflammation to ultimately lead to impaired cognition [14].

#### 3.2.2. AT2R and MasR

AT2R is also undetectable in healthy microglia, but rapidly upregulated as part of a pro-inflammatory response. AT2R is usually upregulated with AT1R as a compensatory mechanism but this paired upregulation has been shown to be blunted in aging [18]. MasRs, on the other hand, are detectable in healthy microglia [28,60]. AT2R and MasR activation facilitate enhanced cognition, cell survival, synaptic maintenance and have both antioxidant and anti-inflammatory properties. Cell surface, mitochondrial and nuclear signaling suppressed ROS induced by NOX. Additionally, nuclear activation of AT2Rs leads to the upregulation of AT2Rs and shifts the microglia toward an M2 phenotype. This also leads to the production of anti-inflammatory cytokines and the upregulation of phagocytic receptors [60,63]. Additionally, the previously discussed SHP-1 and PP2A activation by neuronal AT2R signaling can lead to downregulated Signal Transducer and Activator of Transcription (STAT) 1 and 3 phosphorylation that can dampen microglia activation.

M2 microglia constitutively produce anti-inflammatory cytokines and upregulate phagocytic receptors to participate in phagocytosis within the cortex, hippocampus and basal ganglia. In the cortex and hippocampus, AT2R and MasR activation downregulated iNOS and inflammatory cytokines CXCL 12, (IL)-1β, IL-6 specific to M1 microglia, and upregulated anti-inflammatory markers such as IL-10 and IL-4 [18,52,60,63,64]. Additionally, upon AT1R and MasR induced M2 microglia polarization, brain-derived neurotrophic factor (BDNF) production was enhanced [14]. The enhanced BDNF production promoted cell survival and synaptic plasticity. AT1R and MasR activation also resulted in the upregulation of proteins such as Ptx3 and CD206 which facilitate phagocytosis [18,60,65]. The phagocytic properties allow for synaptic clearance, which in addition to BDNF production, enhanced synaptic plasticity [65]. All of this taken together suggests that AT2R and MasR mediated activation of an M2 anti-inflammatory phenotype may be a mechanism that alleviates cell death and inflammation to ultimately lead to enhanced cognition [14]. When dysregulated this can lead to impaired cognition.

### 3.3. Astrocytes and Oligodendrocytes

Astrocytes are the notorious support cells of the brain. They are involved in a multitude of functions including supplying metabolic support to neurons, maintenance of the BBB, regulation of ion and neurotransmitter concentrations, synaptic transmission and long-term potentiation. Astrocytes also produce the majority of the central angiotensinogen and constitutively secrete it into the extracellular matrix. Oligodendrocytes myelinate neurons and are essential for synaptic transmission. Astrocytes and oligodendrocytes have AT1Rs and AT2Rs expressed on the cell surface [29,52,66]. Astrocytes additionally have MasRs expressed on the cell surface and AT1Rs, AT2Rs and MasRs are expressed on the mitochondria and the nucleus [28,59]. AT4Rs have not been found to be on oligodendrocytes, while the evidence for AT4Rs on astrocytes is inconclusive.

#### 3.3.1. AT1R and ACE

Hyperactivation of AT1R activation and ACE signaling in astrocytes exacerbates cognitive impairment, cell death, inflammation, yet enhances BBB maintenance [18]. AT1R induced inflammation leads to demyelination of oligodendrocytes, which decreases synaptic transmission and impairs neuronal communication. AT1Rs are mainly concentrated on the cell surface [44,49]. In general, AT1R and ACE signaling activates the NOX axis leading to increased ROS production resulting in oxidative stress, similar to that observed in neurons and microglia [28]. Astrocytic foot processes significantly contribute to the BBB and regulate the leukocyte infiltration via chemokine expression [67]. Surprisingly, astrocytic AT1R activation restricts BBB permeability and stabilizes tight junctions [67,68,69].

The cascade of oxidative stress activated by AT1R activation and ACE signaling is a major mechanism which can exacerbate cell death and inflammation, as well as prevent the entry of peripheral immune cells in regions key to cognitive function such as the cortex, hippocampus and basal ganglia. In addition to the direct effects of astrocytic specific AT1R activation the production and excretion of angiotensinogen can lead to increased Ang II to bind to AT1R and exacerbate dopaminergic cell death [70]. In rodents, AT1R activation induced astrogliosis and cell death through ROS production and the effect was enhanced with age [71,72]. Surprisingly, AT1R activation in astrocytic foot processes at the glia limitans specifically enhanced the BBB through threonine-phosphorylation of the tight junction protein occludin and its mobilization to lipid raft membrane microdomains [67,69]. The benefit of the AT1R astrocytic induced maintenance of the BBB and restriction of peripheral immune cell entry depends on the cause and time course of upregulation. In conditions such as stroke, peripheral immune cell entry is beneficial in the acute phase as the innate response to resolve cellular damage. In this context, this BBB maintenance would not be beneficial. Alternatively, sustained inflammation and peripheral immune cell infiltration could lead to the development of cognitive impairment and the restricted access would be beneficial in this context. Within the context of astrogliosis, reactive astrocytes can further compound neuronal and microglia induced inflammation via the activation of ROS, astrocyte cell death can contribute to gaps in the BBB. Perhaps AT1R induced enhancement of the BBB is a compensatory mechanism for the enhanced cell death. Ultimately, AT1R signaling in astrocytes and oligodendrocytes can lead to impaired cognition.

#### 3.3.2. AT2R, MasR and ACE2

The AT2R activation in astrocytes promotes solute leakage [67]. While MasR activation and ACE2 expression enhances cognition via both antioxidant and anti-inflammatory properties [51,73,74]. In oligodendrocytes, AT2Rs promote re-myelination [75]. The exact location of AT2Rs in oligodendrocytes has not been investigated. In astrocytes, AT2Rs are almost exclusively concentrated at the nuclear and mitochondrial levels until upregulation upon injury. The majority of MasRs are located at the mitochondrial level [44,49]. MasRs have a high intracellular density with the main function of regulating mitochondrial and nuclear superoxide production [28].

The antioxidant and anti-inflammatory effects of MasR activation can contribute to neuroprotection and alleviate impaired cognition in regions key to cognitive function such as the cortex, hippocampus and basal ganglia. MasR activation or ACE2 overexpression after injury is associated with a dampening of the inflammatory response and astrogliosis, correlating with improved Morris Water Maze performance [51,73,74]. AT2R signaling in oligodendrocytes prevented demyelination through down regulation of inflammation. AT2R activation also promoted re-myelination to enhance synaptic transmission. AT2Rs induced solute leakage in astrocytes but there are no published effects on ROS or inflammation. Interestingly, the presence of AT2R is required for Ang (1–7), the primary MasR ligand to exert an effect. Reversely, the presence of MasRs are also required for beneficial effects of AT2R activation in astrocytes [29]. This suggests a synergistic interplay between receptors and ligands for AT2R and/or MasR induced downregulation of inflammation, which along with the enhanced synaptic transmission may lead to enhanced cognition, and may impair cognition when dysregulated.

#### 3.3.3. AT4R

AT4Rs are believed to reside on astrocytes but very little evidence of such expression exists [76]. The receptors were shown to be on astrocytes within astrocytic cell cultures and application of Ang IV was shown to increase ERK1/2 signaling in primary astrocytic cultures from rodents [76]. Yet, AT4Rs have not been reported to be observed in vivo, in fact IRAP receptors were found to be absent in the hippocampus of the rodent brain [77]. Another report found that increased astrocytic IRAP expression was associated with normal ageing processes in the rodent brain [78]. Readers can refer to Figure 3 for a summary of receptors signaling found in each cell type.

## 4. Cognitive Benefits of RAS Modulators in PreClinical Animal Models

Angiotensin system modulators have been shown in multiple studies to improve cognitive function [6,79,80]. In this section, we will present selected examples of angiotensin modulators with a positive impact on cognitive impairment of different etiologies. In addition, possible mechanisms and novel drug targets will be discussed and depicted in Figure 2.

### 4.1. AT1R Blockers (ARBs)

#### 4.1.1. Telmisartan

For example, the AT1R blocker, telmisartan, improved memory impairment in a model of repeated cerebral ischemia [81], in a model of chronic cerebral hypoperfusion [82], in a model of acute and chronic stress [83], in LPS-induced neuroinflammation [84], and in uremic mice with chronic kidney disease [85]. Also, treatment of stroke prone spontaneously hypertensive rats (SP-SHR) with telmisartan improved spatial learning and reference memory [86]. The latter effect was due to upregulation of BDNF and its receptor, tyrosine kinase B (trkB), in the hippocampus, which was partially reversed by co-treatment with an antagonist of the transcription factor, PPARγ [86]. Interestingly, telmisartan attenuated diabetes-induced increases in BBB permeability through PPARγ activation to improve diabetes-induced cognitive decline [87]. This indicates that the activation of PPARγ could partially explain the positive impact of telmisartan on cognition. 

#### 4.1.2. Valsartan 

The central cholinergic system has been shown to be instrumental for memory and learning, and deficiencies in cholinergic function are the hallmark of observed cognitive disorders such as AD [88,89,90]. Centrally administered Ang II has been shown to negatively affect learning and memory in rodents, possibly through inhibition of the release of acetylcholine, leading to a decrease in acetylcholine in the synaptic cleft [91,92,93,94]. This suggests that antagonizing central Ang II could improve cognition in part through modulation of cholinergic system.

Blocking the AT1R by valsartan resulted in improvement in forced-swim stress and scopolamine-induced amnesia [95]. The anti-stress effect was associated with a decrease in urinary metabolites of stress-associated precursor molecules such as noradrenaline and cortisone, while the nootropic effect (enhancing learning and memory) was associated with increased acetylcholine through a decrease in acetylcholinesterase activity [95]. In fact, the improvement in memory and learning by valsartan could also be mediated in part through their anti-stress effects because exposure to chronic stress and glucocorticoid administration impairs cognition [95,96].

#### 4.1.3. Losartan

Losartan is another AT1R blocker that has been used in AD animal models. Losartan has been shown to normalize spatial learning and memory capacity in aged mice in the amyloid-β precursor protein (APP) model of AD [6]. Interestingly, losartan reduces astrogliosis and normalizes the expression levels of the AT1R and the AT4R [97]. Chronic blockade of the AT1R increases the production of Ang IV. Overexpressed Ang IV can activate AT1R signaling. When AT1R is blocked Ang IV, even in high concentration, binds solely to AT4R receptors. Ang IV/AT4R signaling increases the concentration of Ang III to bind to activate AT2Rs, NOS to exert anti-inflammatory effects, and enhances synaptic transmission and LTP. It is reasonable to suggest that the effects of losartan, and possibly other ARBs, could also be mediated by facilitating the activation of the AT4R leading to enhancing cognition [98]. Indeed, concurrent administration of losartan with an AT4R antagonist reversed losartan’s beneficial effects on spatial learning and memory [99].

In addition to the positive cognitive effects of losartan in models of AD, losartan shows promising results in cognitive impairment associated with other neurological diseases, such as epilepsy. The cognitive impairment associated with epilepsy is exacerbated by high frequency seizures [100,101]. Losartan improved status epilepticus-induced cognitive impairment in lithium pilocarpine-induced status epilepticus in rats [102]. The mechanism includes suppression of microglial-mediated inflammation and attenuation of hippocampal neuronal loss [74]. Since microglia are the immune cells of the brain and upon activation toward an M1 phenotype they can produce pro-inflammatory cytokines that block neuronal differentiation, they can induce neuronal cell death and brain injury [14,15].

#### 4.1.4. Candesartan

Candesartan is another AT1R blocker that is studied extensively in disease models associated with cognitive impairment. Candesartan ameliorated cognitive impairment induced by long-term repeated amphetamine administration [103], chronic restraint stress [104] and controlled cortical impact [72]. The latter effect is correlated with a reduction in lesion volume and conservation of the hippocampus [72]. Similarly, in earlier studies from our group, candesartan, administered at reperfusion after transient middle cerebral artery occlusion (tMCAO) reduced infarct size and promoted functional recovery both acutely and long-term, indicating a neuroprotective and a neurorestorative effect [105]. The mechanism includes enhancement of angiogenesis through the increased expression of VEGF and angiopoetin-1, neurorestoration through the increased expression of synaptophysin and PSD-95, and the increased expression of BDNF and TrkB [105,106]. Interestingly, knocking-down BDNF by Intracerebroventricular injection (ICV) lentiviral particles abrogated the beneficial effects of candesartan [107]. In addition, candesartan enhanced angiogenesis in vitro in human cerebromicrovascular endothelial cells (hCMEC/D3) both under normoxia and oxygen and glucose deprivation (OGD) [106]. Treatment of primary cortical neurons with conditioned media from candesartan-treated endothelial cells, resulted in improvement in survival and decreased caspase-3 cleavage after OGD [106]. This indicates that candesartan exerts a protective paracrine effect on neuronal cells in addition to the direct angiogenic effects on endothelial cells. In line with this our lab has shown that the angiogenic effect of candesartan was abolished by neutralization of BDNF in vivo [108]. This data suggests that the neurorestorative effects of candesartan could contribute to amelioration of cognitive impairment in different disease models, including stroke. 

### 4.2. Enzyme Inhibitors

#### 4.2.1. Renin Inhibitors

Blocking the AT1R by inhibition of renin (the rate-limiting step in the RAS system) by aliskiren, Renin inhibitors, such as aliskiren, block the catalytic effects of renin but not PRR signaling. Yet, ICV injection ameliorated brain damage and cognitive deficits by augmenting the signaling induced by the Ang II/AT1 axis in a model of subcortical vascular dementia induced by bilateral common carotid artery stenosis [11]. Aliskiren also resulted in improvement in forced-swim stress and scopolamine-induced amnesia alone and in combination with valsartan [95]. This was associated with a decrease in urinary metabolites of stress-associated precursor molecules and increased acetylcholine through a decrease in acetylcholinesterase activity [95]. 

#### 4.2.2. ACE Inhibitors

Transgenic mice with three copies of ACE genes have been used previously to characterize the RAS system [109]. These transgenic mice exhibit high ACE activity with no changes in blood pressure, and impairment in short and long-term memory, as measured by novel object recognition (NOR) testing [109,110]. Impairment in NOR could be generally attributed to perturbations in the dopamine and glutamate systems, or due to antagonism of the muscarinic acetylcholine system, suggesting a link between these systems and RAS [109]. Inhibition of ACE with centrally acting inhibitors such as perindopril reduced cognitive impairment in animal models of AD and VCI [106,109]. The mechanism of the improvement in cognition with ACE inhibition could be explained by multiple factors. For example, the interplay between ACE and dopaminergic neurotransmission or reduction of acetylcholinesterase activity and expression, increased cerebral blood flow, reduced oxidative and nitrative stress, reduced amyloid beta deposition or a combination of these factors [82,109,111]. Another ACE inhibitor that has been studied in cognitive impairment is ramipril. Administration of ramipril, prevented radiation-induced cognitive impairment [112]. The effect was associated with decreased microglial activation in the dentate gyrus, and increased plasma but not cerebral Ang (1–7) [112].

### 4.3. AT2R Agonists

Our lab and others have shown that the effects ARBs, including candesartan, are mediated through the blocking of the AT1R which leads to the activation of the AT2R due to the increased amount of unbound Ang II able to bind to the AT2R [108]. In the context of the AT2R and cognition, direct signaling of the AT2R enhanced spatial memory and prevented cognitive decline after ICV injection of Aβ (1 to 40) in a model of AD [113]. In addition, AT2R-KO animals demonstrate cognitive impairment and decreased hippocampal neurogenesis in females [114].

#### Compound **21** (C**21**)

With recent advances in drug discovery, C**21** was developed as a non-peptide, selective AT2R agonist and has been shown by multiple groups to ameliorate ischemic damage in different models of MCAO [65,115,116]. Our lab has shown that a single dose of C**21**, given at reperfusion (in a mechanical model) is neurovascular protective and improved stroke outcome with no effect on blood pressure in rats [117]. The mechanism of the C**21**-mediated protection includes increased IL-10 and enhanced vascular density in the ischemic penumbra [63,117]. Interestingly, C**21** has recently been shown to attenuate early mortality and increase BDNF, TrkB, and the axonal growth and regeneration marker growth-associated protein 43 (GAP-43) after stroke [110].

Our lab has also shown that activation of the AT2R by C**21** prevents cognitive decline in diabetes possibly through increased cerebral blood flow and/or increased acetylcholine release [118]. C**21** prevented cognitive decline in a model of AD partly due to increased cerebral blood flow (CBF) and field-excitatory post-synaptic potential and neurite outgrowth in hippocampal neurons [113]. The improvement in cognitive function was reversed by blocking the bradykinin B2 receptor, suggesting that the increase in CBF contributes to the cognitive effects of C**21** [113]. 

AT2R and MasR signaling is interdependent, blocking of either receptor abolishes the function of the other receptor [119]. Additionally, in a model of radiation-induced cognitive impairment ACE inhibition with ramipril increased plasma Ang (1–7) levels, suggesting a shift toward the beneficial ACE2/Ang-(1–7)/MasR axis [112]. Therefore, it is possible that the effects of RAS modulators may be due to an indirect effect on the MasRs. A limitation in the development of Ang (1–7) as a therapeutic for MasR stimulation is the large size of the molecule, and its inability to cross the BBB. It also has a very short half-life of around three seconds. Due to these limitations, Ang (1–7) analogs are currently in development.

## 5. Mechanisms of Neuroprotection

### Brain-Derived Neurotrophic Factor (BDNF)

BDNF is a major neurotrophin found in all cell types and is associated with enhanced cognition. The neuroprotective effects of AT1R blockers and AT2R agonists are associated with increased BDNF release [105,107]. Modulation of the angiotensin system to enhance BDNF production may improve cognition by promoting cell survival and exerting antioxidant and anti-inflammatory effects. BDNF promotes neuronal survival, morphogenesis, synaptic plasticity and neurotransmitter release [120,121]. In microglia, BDNF promotes synaptic plasticity through pruning as well as learning-related synapse formation [122]. It also promotes polarization of microglia toward a M2 phenotype and down regulates pro-inflammatory cytokine and ROS production. Upon M2 polarization, microglial-derived BDNF production increases. 80% of microglial-derived BDNF production is from AT2R positive M2 microglia and it mediates wound healing while further downregulating inflammation through promoting the production of anti-inflammatory cytokines [65]. In astrocytes, AT2R mediated release of BDNF suppresses astrogliosis [123]. Taken together, this suggests that modulation of the angiotensin system to enhance BDNF production may be a key target in cognitive impairment.

## 6. Translational and Clinical Implications

The literature is replete with clinical investigations suggesting an association between AT1R blockade and improved cognitive function. Clinical studies have demonstrated an increased AT1R expression in the post-mortem cortex of AD patients in comparison to non-disease control patients [124]. AT1R expression has also been shown to impact dopaminergic neurons within the basal ganglia of PD patients, where it is upregulated in aged individuals and is associated with neuroinflammation and NOX induced ROS [18]. Studies evaluating the impact of ARBs on white matter hyperintensities (WMH) within the hippocampus of patients demonstrated that ARB treatment was associated with less WMH volume and better memory performance [125]. Since WMHs are a common indicator of axonal demyelination and cognitive impairment, this suggests that repression of AT1R signaling could potentially improve synaptic transmission and cognitive function [126].

One of the largest systematic reviews done in recent years, however, revealed the difficulty in proving a superior protective effect when blood pressure lowering itself, regardless of agent, appears to reduce all-cause dementia by 9% [127]. An important caveat is that significant protection was only seen when the randomized trials were combined with observational studies. Including data from over 850,000 individuals in 30 studies, ARBs were associated with less cognitive decline and were significantly better than either diuretics, beta blockers or angiotensin converting enzyme (ACE) inhibitors [127]. Subsequently, a large group of 1626 individuals (55–91 years of age), either cognitively normal or with mild cognitive impairment (MCI) were followed for 3 years for changes in cognition and imaging evidence of brain injury. As stated previously, treatment of hypertension with ARBs that were known to cross the blood brain barrier (telmisartan, candesartan, valsartan) were associated with preserved cognition and reduced WMH volume, compared to patients treated with all other agents [2]. Perhaps even more convincing was a report that, of 784 patients with MCI and hypertension (mean age 74 years), treatment with RAS medications (either ARBs or ACE inhibitors) was associated with a significant reduction in conversion to AD (33% vs. 40%) at 3 years, compared to other antihypertensive agents [128]. This difference existed despite a higher mean blood pressure in the RAS medications group [128]. In fairness, other smaller studies in selected populations have shown unique benefits of diuretics [129] and calcium channel blockers [130] in the prevention of cognitive impairment and dementia. In these studies, however, the use of RAS medications were limited, decreasing the power to detect a difference if it did exist. It is likely that the benefit of ARBs and ACE inhibitors on cognitive impairment involves blood pressure lowering in addition to centrally mediated effects on inflammation, apoptosis and preservation of cerebral blood flow [42,131,132,133].

To date, there are no clinically available compounds that specifically activate AT2Rs, AT4Rs or MasRs, without causing changes in blood pressure. The specific AT2R receptor agonist, C**21**, is currently in clinical development for pulmonary fibrosis and holds promise in central nervous system disorders [134]. Whether the cognitive benefits seen in preclinical models will translate to the human condition remains to be proven.

## 7. Concluding Remarks

In conclusion, it can be inferred that RAS modulators ameliorate cognitive impairment associated with different disease models through highly overlapping mechanisms. Targets for improvement of cognitive impairment include the Ang IV/AT4R (IRAP) axis, ACE2/Ang-(1–7)/MasR axis, and/or Ang II/AT2R axis. It is intriguing to harness the beneficial impact of RAS modulators in the fields of post-stroke cognitive impairment (PSCI) as a direct application of their neurorestorative effects. In this context, it is important to point out the sex differences in the angiotensin system. Clinically, the incidence of dementia and cognitive impairment is higher in women than in men [135]. Dementia and cognitive impairment is specifically higher in post-menopausal than pre-menopausal women of similar age [136]. Although there are no clinical studies that evaluate the sex or estrogen mediated modulation of the cerebral RAS, preclinical studies show that rodent female brains have more angiotensinogen-positive neurons than males [137] and is further upregulated by estrogen supplementation [138]. Ovariectomy (OVX) is a surgical procedure performed to evaluate the effects of estrogen depletion in rodents. OVX upregulated AT1Rs and ACE and downregulated AT2Rs [61,114,139,140] and was reversed upon estrogen supplementation [139,141]. When comparing intact females to males, only females experienced cognitive deficits and neuronal loss upon AT2R knockout [114] and enhanced ACE2 expression levels upon restoration of AT2R signaling [142]. Interestingly, OVX also upregulated MasRs within the forebrain. The upregulation was further enhanced upon estrogen supplementation [143]. Evidence also suggests that there may be a sexual dimorphism in the interdependency of MasR and AT2R signaling. The physiological effects of MasR activation were blunted when AT2Rs were blocked in females, but there was no impact when blocked in males [144]. More studies evaluating sex differences within the angiotensin system and cognitive impairment are needed. Aging and disease states such as hypertension and diabetes can precipitate a dysregulation of the angiotensin system and can be reviewed here [18,145,146]. More studies are needed to evaluate the modulation of the angiotensin system and its contribution to the increased incidence of cognitive impairment observed in aging, hypertension and diabetes.

Unanswered questions regarding RAS modulation and cognition include, (1) Is AT2R stimulation necessary and sufficient for prevention of PSCI?; (2) How does the AT4R interact with the AT2R after brain injury to promote recovery?; (3) How is it possible that females have higher AT2R expression but are at increased risk of cognitive impairment?; and (4) What is the molecular/cellular mechanism by which AT2R stimulation preserves cognition? Future investigations of varied models of cognitive impairment, will be necessary to answer these questions and move the field toward an effective treatment for patients.
